# A Developmental System Perspective to Interpret the Link between Parental Fixed Mindset and Youth Mental Health: A Moderated Mediation Model

**DOI:** 10.3390/ijerph192013285

**Published:** 2022-10-14

**Authors:** Wei Qi, Jing Shi, Lijuan Cui

**Affiliations:** 1 Institute of Brain and Education Innovation, East China Normal University, Shanghai 200062, China; 2 School of Educational Science, Shenyang Normal University, Shenyang 110034, China; 3 School of Psychology and Cognitive Science, East China Normal University, Shanghai 200062, China

**Keywords:** fixed mindset, core self-evaluation, peer support, mental health, developmental system

## Abstract

From a developmental system perspective, the present study constructed a moderated mediation model to examine whether youth core self-evaluation (individual factor) served as a mediator and peer support (peer system) served as a moderator in the effect of parental fixed mindset (family system) on youth mental health symptoms. In total, 658 pairs of emerging adults and their parents participated in this study. Youth completed measurements on core self-evaluation, peer support, and mental health symptoms, while their parents filled in the questionnaire on fixed mindset. Mediation analysis indicated that parental fixed mindset was related to increased youth mental health symptoms, and youth core self-evaluation partially mediated this relationship. Moderated mediation analysis suggested that peer support mitigated the mediating process with the direct pathway from parental fixed mindset to youth mental health symptoms and the indirect pathway from parental fixed mindset to youth core self-evaluation being weaker at a high level of peer support. This study highlights how and when a family system, peer system, and individual factors combine to influence youth mental health. The findings suggest it is the interaction of these factors that has to be addressed in efforts to reduce the prevalence of youth mental health symptoms.

## 1. Introduction

The transitional period between the end of adolescence and the beginning of full adulthood, typically spanning the ages 18–29, is known as emerging adulthood [1]. In this stage of life, young adults explore a range of life alternatives, including identity, relationships, education, and employment on one hand, and encounter uncertainty and social instability on the other [2]. Emerging adulthood is marked by greater independence than in adolescence, but less stability than in maturity [3], which leads to particularly common mental health symptoms during this period. About 47% of young adults report having suffered a mental disorder in the previous year [4]. In fact, the rate of mental illness among young adults is higher than among any other adult age group [5]. Anxiety disorders and major depressive episodes are the most common types of mental illness among young adults [5]. Although emerging adults are often perceived as leaving their family home and having more social interactions with peers, a lack of true self-sufficiency and full independence means that the family remains a developmental environment that is no less important to young people than their peers [6]. Up to this point, few studies have looked at how the family can influence youth mental health [7], and studies that simultaneously explore the influences of the family and peers are even fewer. From a developmental systems perspective of human development [8], this study aims to explore how the family system (i.e., parental fixed mindset) and peer system (i.e., peer support) jointly impact youth mental health.

### 1.1. Parental Fixed Mindset on Youth Mental Health Symptoms

Mental health symptoms refer to the psychosocial effects of mental health problems, including anxiety, depression, hostility, paranoid ideation, etc. [9]. Among the many factors associated with youth mental health symptoms, the impact that the family has on young people cannot be underestimated [6]. The parent factor, as a crucial factor in the family system, also has a significant impact on young people’s mental health [10]. The parental fixed mindset, as an intrinsic concept possessed by parents, refers to their belief that fundamental human characteristics are fixed and cannot be altered via effort and practice [11]. Social culture and individual attributes could influence the development of mindsets [12,13]. Fixed-mindset parents who avoid stress and challenge raise their offspring with the aim of achieving and performing well rather than focusing on the learning process [14]. Thus, whether by shaping parent–child dynamics, the broader family environment, or offspring’s own beliefs, parental fixed mindset may affect mental health in their offspring. Various research has demonstrated the detrimental effect of fixed mindset on individuals’ mental health. For example, youth with a fixed mindset are at increased risk for mental health symptoms [15]. In addition, fixed mindset was associated with future psychological distress, less resilience, and more psychiatric symptoms [16,17,18]. To date, while many studies have explored how a fixed mindset might affect individuals’ mental health, very few studies have looked at how a parental fixed mindset can affect their offspring’s mental health within family system. Based on established findings, this study proposes Hypothesis 1.

**Hypothesis** **1.** *Parental fixed mindset would positively predict youth mental health symptoms*.

### 1.2. Youth Core Self-Evaluation as a Mediator

As for the mechanisms underlying the influence of fixed mindset on mental health, existing research is limited at the individual level, let alone based on a family system. This study sought to explore the role of youth core self-evaluation as a mediator between parental fixed mindset and mental health symptoms. On one hand, parental fixed mindset as a cognition or belief system in parents may influence core self-evaluation in their newly adult children. Core self-evaluation, comprised of four dimensions (locus of control, emotional stability, generalized self-efficacy, and self-esteem), refers to a deeper assessment of a person’s value, efficacy, and competence [19]. In the socialization process, individuals form their cognitive patterns by accepting and internalizing their parents’ ideas, values, and norms, which in turn shape their perceptions and evaluations of themselves, others, and events [7]. It can be argued that parents shape their offspring’s beliefs through parent-child interactions [20]. Moreover, a fixed mindset has been found to influence self-esteem [21] and self-efficacy [22], which are two dimensions of core self-evaluation. Thus, youth core self-evaluation may be influenced by a parental fixed mindset. On the other hand, core self-evaluation significantly predicts mental health symptoms at the individual level [23]. Prior studies have confirmed a negative core self-evaluation is highly related to a wide range of mental health symptoms, including depression [24], burnout [25], emotional exhaustion [23], and suicidal ideation [26]. Therefore, this study proposes Hypothesis 2.

**Hypothesis** **2.** *Youth core self-evaluation would act as a mediator between parental fixed mindset and youth mental health symptoms*.

### 1.3. Peer Support as a Moderator in the Mediation Process

Peer support refers to individuals providing mutual support, discussing their problems, and receiving empathy and suggestions from their peers [27]. Resilience research suggests risk factors are those that make individuals more vulnerable to adversity and lead to poor developmental outcomes, while protective factors are those that mitigate the negative impact of adversity on individuals and lead to desirable developmental outcomes [28]. As a fixed mindset is generally associated with less resilience and other negative outcomes [16,17,18], a parental fixed mindset can be deemed as a risk factor. As the stress buffering model asserts that relationships may be protective to health by being a source of support when coping with stress [29], peer support can be interpreted as a protective factor. According to a developmental systems perspective of human development, individual development is not only influenced by different developmental systems alone, but also by the interaction between these developmental systems [8]. Taken together, the adverse effect of a risk factor in a family system (i.e., parental fixed mindset) on youth mental health may be mitigated by a protective factor in a peer system (i.e., peer support). In early adulthood, peers take on a crucial role in providing emotional and social support [30]. Peer support has been proven to be act as a protective role in reducing mental health risks due to previous studies showing that it considerably attenuated the adverse effect of enacted stigma on psychological distress [31]. Moreover, another study showed that the unfavorable effect of negative parenting on externalizing behavior was only significant in adolescents with low peer group affiliation [32]. These studies illustrate that the positive peer system may help emerging adults defuse risk factors in other developmental systems and maintain their mental health. Therefore, this study proposes Hypothesis 3.

**Hypothesis** **3.** *Peer support would moderate the direct pathway from parental fixed mindset to youth mental health symptoms, attenuating the effect of parental fixed mindset in boosting youth mental health symptoms*.

Similarly, from a developmental systems perspective [8], the interaction of the peer system and family system may influence youth self-cognition as well. In addition to the family, the socializing influence of one’s peers endures at least until one’s twenties [33]. Peer support has been found to increase self-esteem [34] and self-efficacy [35], which are two indicators of core self-evaluation. On the contrary, it has been found that the adolescence to adulthood transition is hampered by social isolation and the loss of one’s peer group [36]. These studies suggest that peer support as a positive factor in peer system contributes to the development of self-cognition, such as core self-evaluation, in emerging adults. Therefore, this study proposes Hypothesis 4.

**Hypothesis** **4.** *Peer support would moderate the first stages of the mediation procedure, buffering the negative pathway from parental fixed mindset to youth core self-evaluation*.

Finally, the Person-Context Interaction Theory deems that an individual’s psychology and behavior develop through the interaction of personal and environmental factors [37]. For example, one study found that youth negative emotion is influenced by the interaction of peer system and individual factors [38]. Thus, youth core self-evaluation (an individual factor) may interact with peer support (a factor in peer system) and have an impact on youth mental health symptoms. Therefore, this study proposes Hypothesis 5.

**Hypothesis** **5.** *Peer support would moderate the second stage of the mediation procedure, mitigating the negative pathway from youth core self-evaluation to youth mental health symptoms. Taken together, it is assumed that the direct and indirect effects of parental fixed mindset on youth mental health symptoms are mitigated by peer support*.

### 1.4. The Present Study

**Figure 1:**
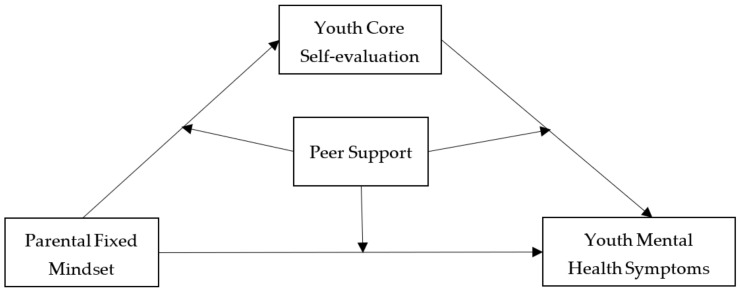
Hypothetical model.

From a developmental system perspective, this research sought to simultaneously investigate the effects of parental fixed mindset (family system), peer support (peer system), and youth core self-evaluation (individual factor) on youth mental health symptoms. Specifically, this study constructed a model with youth core self-evaluation as a mediator and peer support as a moderator to interpret the effect of parental fixed mindset on youth mental health symptoms (see Figure 1).

## 2. Materials and Methods

### 2.1. Participants

Emerging adults (ages 18–29) and their parents were recruited for this study. After excluding questionnaires with lots of missing or repeated answers, 658 valid pairs of questionnaires were received. Among the youth participants, 252 were males and 406 were females, M_age_ = 22.14 years, and SD_age_ = 1.37 years. Among the parent participants, 215 were fathers and 443 were mothers, M_age_ = 49.68 years, and SD_age_ = 3.02 years. 

### 2.2. Measures

#### 2.2.1. Fixed Mindset

The Domain-General Mindset Scale [11] was employed to evaluate fixed mindset in parents. A Likert scale with 6 points was used to evaluate the 3 items, in which 1 implies total disagreement and 6 implies total agreement. A higher score signifies that parents have a more fixed mindset. The Cronbach’s α was 0.84 for this scale.

#### 2.2.2. Core Self-Evaluation

Youth core self-evaluation was assessed employing the Core Self-Evaluation Scale [39]. This scale has 12 items based on four dimensions: emotional stability, generalized self-efficacy, self-esteem, and locus of control. A Likert scale with 5 points was used for response, with 1 signifying total disagreement and 5 signifying total agreement. More positive core self-evaluation is denoted by higher scores. The Cronbach’s α was 0.82 for this scale.

#### 2.2.3. Peer Support

Youth peer support was assessed using an abridged version of the Social and Emotional Loneliness Scale [40]. This scale comprises five items. A Likert scale with 7 points was used for response, with 1 signifying total disagreement and 7 signifying total agreement. After scores of negatively-worded items were reversed, better peer support is denoted by higher scores. The Cronbach’s α was 0.79 for this scale.

#### 2.2.4. Mental Health Symptoms

The Depression Anxiety and Stress Scale 21 was adopted to assess youth mental health symptoms [41]. There are a total of 21 items on this scale, with 7 each measuring depression, anxiety, and stress. A Likert scale with 4 points was used for response, in which 0 implies never and 3 implies always. More severe mental health symptoms are shown by higher scores. The Cronbach’s α was 0.90 for this scale.

### 2.3. Procedure

This study gained approval from the first author’s University Ethics Committee. The emerging adults and their parent clicked on different questionnaire links to finish their respective measurements via an online survey platform. Before data collection, participants first read the informed consent form following the assurance of confidentiality and anonymity. Participants would not begin the survey until they have clicked the “Agree” button at the bottom of the informed consent form. The young adults in this study filled out evaluations on core self-evaluation, peer support, and mental health symptoms, while their parents completed the measurement on fixed mindset. Both questionnaires were matched by the same invitation code. When completing the survey, all participants were appreciated.

### 2.4. Data Analysis

First, common method bias, descriptive statistics, and Pearson correlations were estimated by SPSS 24.0 (SPSS Inc., Chicago, IL, USA). Second, to examine the moderated mediation model of central interest, Model 4 and Model 59 from the PROCESS macro [42] were deployed, with 95% confidence interval (CI) evaluated by 5000 bootstrapped samples. Finally, the essence of the moderating effect was uncovered by simple slope analyses. All study variables were mean-centered in Model 4 and Model 59 before data analyses.

## 3. Results

### 3.1. Preliminary Analyses

This study first utilized Harman’s single factor test to examine common method bias [43]. The results indicated that 12 factors had eigenvalues above 1, and 19.84% of the total variation was attributed to the first factor, below the threshold of 40%. Thus, this study discovered no evidence of common method bias.

Then, for the primary variables, descriptive analyses and Pearson correlations were conducted (see Table 1). Correlation analysis showed that parental fixed mindset and youth mental health symptoms were positively correlated (*r* = 0.17, *p* < 0.01), which supported Hypothesis 1. In addition, main variables were significantly correlated with each other, except for the relationship between parental fixed mindset and peer support (*r* = 0.08, *p* > 0.05). 

### 3.2. The Mediation Model Analysis

Table 2 and Table 3 exhibited the results of the mediation model analysis conducted with PROCESS macro Model 4 [42]. Taking youth gender and age as covariates, parental fixed mindset negatively predicted youth core self-evaluation (β = −0.12, *p* < 0.05). When parental fixed mindset and youth core self-evaluation simultaneously predicted youth mental health symptoms, both parental fixed mindset (β = 0.11, *p* < 0.05) and youth core self-evaluation (β = −0.36, *p* < 0.001) turned out significant predictors of youth mental health symptoms. Moreover, as Table 3 displayed, the indirect effect of youth core self-evaluation was 0.04, 95% CI = [0.01, 0.09]. The direct effect of parental fixed mindset on youth mental health symptoms was 0.11, 95% CI = [0.03, 0.21]. The Bootstrap 95% CI for the direct and indirect pathways did not contain 0, indicating that both of them reached a significant level. Thus, the connection between parental fixed mindset and youth mental health symptoms was partially mediated by youth core self-evaluations. Hypothesis 2 was supported.

### 3.3. The Moderated Mediation Model Analysis

**Figure 2:**
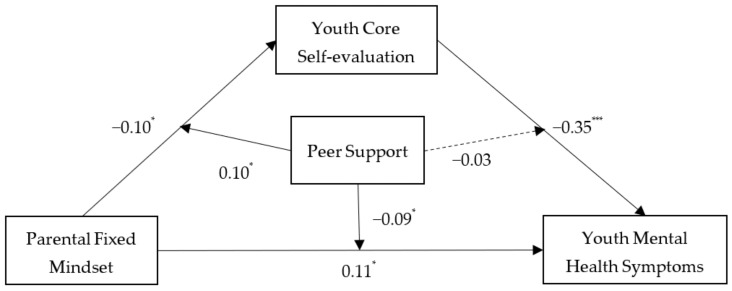
The moderated mediation pathways. * *p* < 0.05, *** *p* < 0.001.

Table 4 and Figure 2 showed the results of the moderated mediation analysis conducted with PROCESS macro Model 59 [42]. Taking youth gender and age as covariates, the interaction of parental fixed mindset and peer support had significant effects on youth core self-evaluation (β = 0.10, *p* < 0.05) and youth mental health symptoms (β = −0.09, *p* < 0.05). However, the interaction of youth core self-evaluation and peer support did not have a significant effect on youth mental health symptoms (β = −0.03, *p* > 0.05). These findings indicated that peer support moderated the link between parental fixed mindset and youth core self-evaluation and between parental fixed mindset and youth mental health symptoms. 

**Figure 3:**
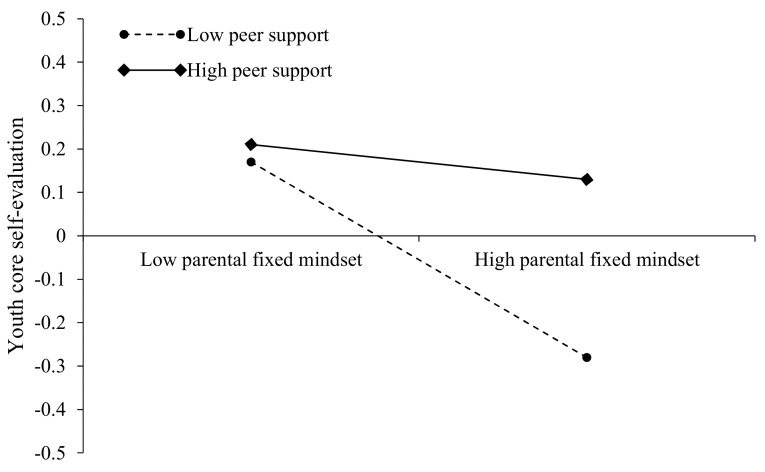
Interaction between parental fixed mindset and peer support on youth core self-evaluation.

**Figure 4:**
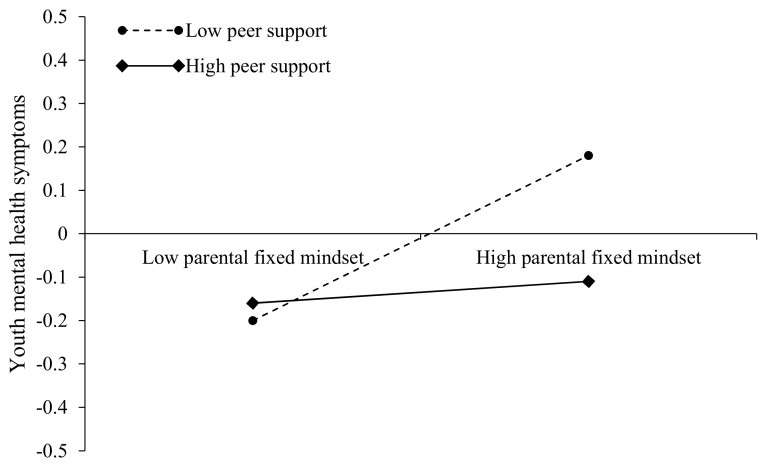
Interaction between parental fixed mindset and peer support on youth mental health symptoms.

Finally, the nature of the moderating effect was further elucidated by simple slope analysis. As for the interaction of parental fixed mindset and peer support on youth core self-evaluation (see Figure 3), the negative effect of parental fixed mindset on youth core self-evaluation was significant for those with low peer support (1 SD below the mean; β_simple_ = −0.30, *p* < 0.001), but nonsignificant for those with high peer support (1 SD above the mean; β_simple_ = −0.06, *p* > 0.05). As to the interaction of parental fixed mindset and peer support on youth mental health symptoms (see Figure 4), the positive effect of parental fixed mindset on youth mental health symptoms was significant for those with low peer support (1 SD below the mean; β_simple_ = 0.22, *p* < 0.01), but nonsignificant for those with high peer support (1 SD above the mean; β_simple_ = 0.04, *p* > 0.05). Therefore, these results provided support for Hypothesis 3 and 4, but not for Hypothesis 5.

## 4. Discussion

By developing a moderated mediation model, this study investigated how parental fixed mindset influences youth mental health symptoms and when the influence is buffered. As hypothesized, our results showed that the connection between parental fixed mindset and youth mental health symptoms was partially mediated by youth core self-evaluation. Peer support moderated two pathways in the mediation process, that is, the direct pathway from parental fixed mindset to youth mental health symptoms and the first half of the mediation procedure from parental fixed mindset to youth core self-evaluation. However, this study found no support for peer support as a moderator in the second half of the mediation procedure from youth core self-evaluation to youth mental health symptoms. 

### 4.1. Youth Core Self-Evaluation as a Mediator

This study demonstrates that parental fixed mindset is linked to youth mental health symptoms via a partially mediating role of youth core self-evaluation, consistent with what was previously documented. Firstly, previous studies suggested that having a fixed mindset is harmful to one’s mental health. For example, a fixed mindset was associated with less resilience, future psychological distress, and more psychiatric symptoms [16,17,18]. Secondly, parental attitudes tend to influence offspring’s self-cognition, including self-esteem [21] and self-efficacy [22]. Finally, core self-evaluation as an individual cognitive factor has been shown to predict mental health symptoms, including depression [24] and suicidal ideation [26]. Therefore, our finding that a parental fixed mindset leads to more youth mental health symptoms by weakening core self-evaluation is congruent with the existing literature. To be noted, although extant studies have suggested that family system and the individual’s psychological factor both play a role in shaping a young adult’s mental health [44], it has not fully explored the underlying mechanisms of how family system and individual factors combine to impact youth mental health. This study found that parental fixed mindset resulted in worse youth mental health by diminishing youth self-evaluation, which revealed the specific process by which family system and individual factors jointly influence youth mental health.

### 4.2. Peer Support as a Moderator

Moreover, the results showed that peer support moderated the influence of parental fixed mindset on youth mental health symptoms. For the direct pathway in the mediation procedure, the positive association between parental fixed mindset and youth mental health symptoms became nonsignificant at a high level of peer support. Such results are in line with research in the peer support field. High levels of peer support imply effective companionship, mutual help, and emotional communication [38]. A prior study found that peer support may contribute to reducing the negative impacts of stressors on mental health, in addition to directly safeguarding mental health [45]. For example, a study of young people who identify as sexual minorities revealed that friendships’ support for particular sexual orientations weakened the link between minority stress and emotional distress [46]. It should be mentioned that the majority of prior studies in the peer support field have underlined the protective role of peer support from the perspective of person-context interaction. This study, however, reveals the importance of the interaction between family system and peer system in maintaining youth mental health from the developmental systems perspective.

Additionally, peer support moderated the first half of the mediation procedure. Specifically, parental fixed mindset had a significantly negative association with youth core self-evaluation at a low level of peer support, but not at a high level of peer support. There may be two main reasons for such results. First, peer support increases an individual’s social and psychological capital to cope with risk factors [47]. High peer support means that individuals not only receive more social support from friends [48] but it also enhances an individuals’ cognitive resources, such as psychological resilience [49], thus contributing to a reduction in negative outcomes caused by risk factors. Second, high peer support also provides youth with access to a supportive and encouraging environment that enhances self-efficacy [22]. Such a positive peer system contributes to changing the negative implication of parental fixed mindset that individual abilities cannot be changed, making the unfavorable influence of a negative family system on youth core self-evaluation less effective. Thus, peer support’s role in the association between parental fixed mindset and youth core self-evaluation similarly suggests that there is an interaction between family system and peer system, which can jointly influence the psychological development of youth.

However, peer support did not moderate in the second half of the mediation process. Such results may be due to youth core self-evaluation’s stable predictive power for youth mental health symptoms. That is, even high levels of peer support did not significantly enhance youth core self-evaluation’s protective power for mental health symptoms. Since the effect of an individual factor on mental health symptoms remains stable in youth, the individual factor turns out to be an aspect worth considering when exploring youth mental health. In addition, the Protective-Protective Model suggests that the effects of two protective factors (e.g., peer support and youth core self-evaluation) on the outcome variable may result in the exclusion hypothesis [50]. The exclusion hypothesis refers to the phenomenon that the effect of one protective factor would be alleviated by another protective factor, thus two protective factors fail to have a more protective effect on the outcome variable than a single one [50]. Thus, the nonsignificant moderating role of peer support in the mediating pathway from youth core self-evaluation to youth mental health symptoms suggests a possible exclusion hypothesis between the two protective factors on the one hand, and youth core self-evaluation’s stable predictive power for youth mental health on the other hand.

### 4.3. Limitations and Implications

Although this study reveals the underlying mechanism by which a family system, peer system, and individual factors combine to influence youth mental health, there are still some limitations. First, this study only examined the effects of parental fixed mindset in the family system and peer support in a peer system on youth mental health symptoms. Future research could investigate the influence of other factors in family and peer systems on individual development (including mental health) to more comprehensively undercover the mechanisms by which these two developmental systems contribute to youth development. Secondly, the cross-sectional design employed for this study greatly hindered it from establishing causal relationships between study variables. For future research, a longitudinal design may be conducted to discover causal evidence for the moderated mediation model. Third, the parental fixed mindset data used in this study did not distinguish between father’s and mother’s, which might have led to the relatively low effect sizes in the moderated mediation model. A study investigating adolescent participants found gender differences in those with a fixed mindset, with girls having a higher level of fixed mindset compared with boys [51]. If gender differences in people with a fixed mindset could also be found in parent subjects, some differences in effect sizes may be observed in the moderated mediation model between the father’s and mother’s groups. Future research could explore whether a father’s fixed mindset and mother’s fixed mindset result in a significant difference in the moderated mediation model. Fourth, OSL regression-based PROCESS macro used in this study has some limitations versus structural equations models, such as assuming fixed effects, continuous outcomes, and the absence of random measurement error [42]. Despite the above limitations, both theoretical and practical implications arise from this study. On one hand, this study fills a critical need to explore how the interaction of different developmental systems impacts youth cognitive factor and subsequent mental health. Thus, this study is a new application of a developmental systems perspective to youth mental health field, which undercovers “how” and “when” the interaction of family system and peer system affects youth mental health symptoms. On the other hand, the attenuating role of peer support in the undesirable link between parental fixed mindset and youth core self-evaluation and mental health elucidates that preventive interventions for youth mental health problems should focus not only on the separate influence of family, peer, and individual factors but also on the combined effect of these factors.

## 5. Conclusions

This study contributes to the extant literature by investigating a moderated mediation model that comprehensively explains how and when critical factors within a developmental system jointly impact youth mental health symptoms. The findings suggest that having a parental figure with a fixed mindset is a risk factor in family systems, and is positively related to youth mental health symptoms. Moreover, youth core self-evaluation as an individual factor mediates the link between parental fixed mindset and increased mental health symptoms in young adults. Furthermore, peer support as a protective factor in peer system moderates the mediating process with the direct pathway from parental fixed mindset to youth mental health symptoms and the indirect pathway from parental fixed mindset to youth core self-evaluation being weaker in the presence of high peer support. Therefore, this study illustrates both “how” an at-risk family system negatively influences youth mental health and “when” a supportive peer system mitigates this negative impact.

## Figures and Tables

**Table 1:** Correlations and descriptive statistics of main variables.

	*M*	*SD*	1	2	3	4
1. Parental fixed mindset	3.51	0.93	1			
2. Youth core self-evaluation	2.14	0.76	−0.14 **	1		
3. Peer support	3.79	0.84	0.08	0.19 **	1	
4. Youth mental health symptoms	1.62	0.71	0.17 **	−0.37 ***	−0.16 **	1

Note. ** *p* < 0.01, *** *p* < 0.001.

**Table 2:** Regression analysis of the mediation model.

Predictors	Step 1 (Youth CSE)				Step 2 (Youth Mental Health Symptoms)			
	*β*	*SE*	95%CI	*t*	*β*	*SE*	95%CI	*t*
Parental FM	−0.12	0.03	[−0.23, −0.04]	−2.58 *	0.11	0.02	[0.03, 0.21]	2.42 *
Youth CSE					−0.36	0.05	[−0.45, −0.26]	−10.04 ***
*R^2^*	0.15				0.22			
*F*	37.94 ***				41.39 ***			

Note. The variables are standardized before being entered into the regression equation. * *p* < 0.05, *** *p* < 0.001.

**Table 3:** Bootstrapping analysis of the mediation model.

	Effect	*SE*	95% CI	Ratio to Total Effect
Direct Effect	0.11	0.02	[0.03, 0.21]	73.33%
Indirect effect	0.04	0.02	[0.01, 0.09]	26.67%
Total effect	0.15	0.03	[0.11, 0.27]	100%

**Table 4:** Analysis of the moderated effect of peer support on the mediation model.

Predictors	Model 1 (Youth CSE)				Model 2 (Youth Mental Health Symptoms)			
	*β*	*SE*	95%CI	*t*	*β*	*SE*	95%CI	*t*
Parental FM	−0.10	0.04	[−0.16, −0.04]	−2.16 *	0.11	0.05	[0.04, 0.018]	2.29 *
Peer support	0.14	0.05	[0.07, 0.20]	2.53 *	−0.19	0.06	[−0.27, −0.13]	−5.76 ***
Parental FM × Peer support	0.10	0.05	[0.03, 0.15]	2.11 *	−0.09	0.04	[−0.12, −0.03]	−2.05 *
Youth CSE					−0.35	0.06	[−0.41, −0.28]	−7.67 ***
Youth CSE × Peer support					−0.03	0.03	[−0.06, 0.01]	−0.85
*R^2^*	0.08				0.19			
*F*	6.24 ***				12.77 ***			

Note. FM = fixed mindset, CSE = core self-evaluation. * *p* < 0.05, *** *p* < 0.001.

## Data Availability

The data presented in this study are available from the corresponding author on reasonable request.
